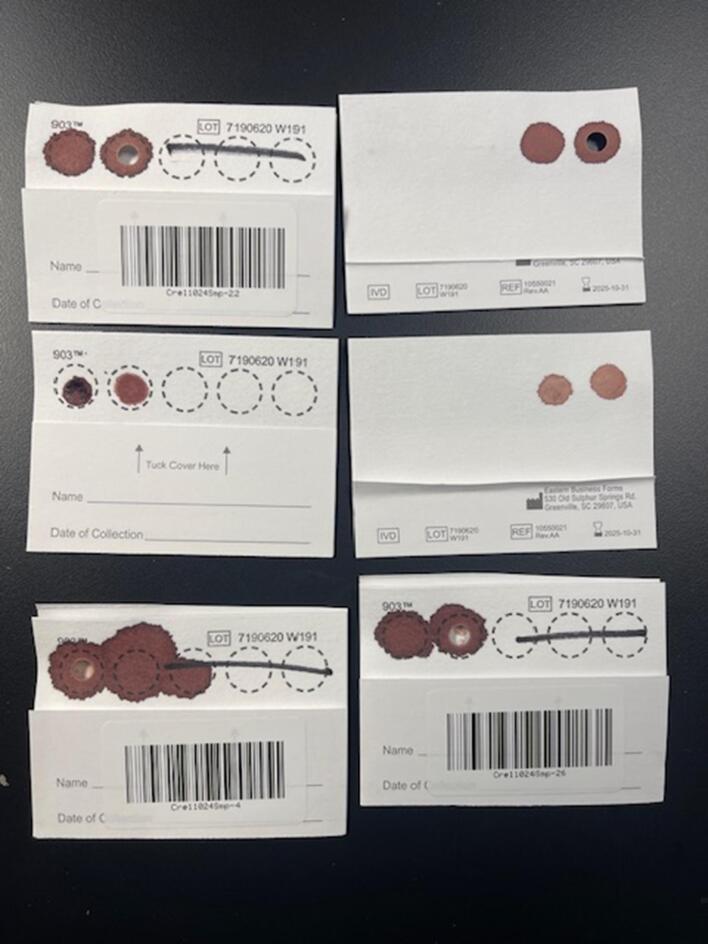# Development and validation of an LC-MSMS method to quantify creatinine from dried blood spots

**DOI:** 10.1016/j.jmsacl.2024.03.001

**Published:** 2024-03-06

**Authors:** Carlos Torres, Rogers A. Muldrow, Anissa R. Naranjo, Steven W. Cotten, Christina C. Pierre, Dina N. Greene

**Affiliations:** a LetsGetCheked, Monrovia, CA, USA; b Department of Pathology and Laboratory Medicine, Chapel Hill, NC, USA; c Department of Pathology and Laboratory Medicine, Penn Medicine Lancaster General Hospital, Lancaster, PA, USA; d Department of Pathology and Laboratory Medicine, Perelman School of Medicine, University of Pennsylvania, Philadelphia, PA, USA; e University of Washington Seattle, Department of Laboratory Medicine and Pathology, Seattle, WA, USA

**Keywords:** Creatinine, Dried blood spots, LC-MS/MS, Chronic kidney disease screening

## Abstract

**Introduction:**

Screening for chronic kidney disease relies on accurate and precise creatinine measurements. Traditionally, creatinine is measured in serum or plasma using high-throughput chemistry analyzers. However, dried blood spots (DBS) can also be utilized to improve testing access.

**Methods:**

Samples were obtained from a 6 mm DBS punch, which was reconstituted in water before undergoing an acetonitrile crash. The resulting supernatant was diluted using an 80:20 acetonitrile: water before injection. Creatinine was identified using an isocratic gradient, and detected using an API 4000 triple quadrupole mass analyzer. Quantification relied on matrix-matched calibrators, with values harmonized to the Roche Cobas enzymatic assay. Validation studies assessing method performance included precision, linearity, accuracy, method comparison, stability, interference, and matrix effects.

**Results:**

The LC-MSMS assay was linear from 0.3 to 20 mg/dL (y = 1.02x-0.11; R^2^ = 0.996). Precision ranged from 5.2 to 8.1 % using matrix-matched controls (n = 4) that spanned the analytical measurement range. LC-MSMS results corresponded to the enzymatic assay (Roche) with a fitted line equation of y = 0.956x–0.07 (R^2^ = 0.995; n = 173). The Siemens and Roche enzymatic assays demonstrated higher accuracy in correlating to the DBS creatinine concentration (n = 40 paired venous/DBS collections) compared to the Beckman Jaffe assay (-2.5 % and −0.8 % versus −6.3 % and −4.1 %, respectively) or the iSTAT (-28.4 % and −27.1 %, respectively). Accuracy was unaffected by hematocrit, blood spot volume, excess IgG or IgA, or hypertriglyceridemia. No matrix effects were observed, and both extraction and processing efficiency were robust.

Ambient stability extended to at least 10 days, and exposure to extreme temperature did not affect the creatinine results.

**Conclusion:**

We successfully developed an accurate and precise LC-MSMS method for quantifying creatinine in DBS.

## Highlights

• Creatinine can be accurately measured from dried blood spots (DBS) using LC-MSMS.
• DBS creatinine measurements were not significantly affected by varying hematocrit or blood spot volume.
• DBS analysis of creatinine has the potential to improve access to CKD screening.

## Introduction

Chronic kidney disease (CKD) continues to be a global issue with millions of people in the US unaware of its onset [1]. Early detection and treatment of CKD can slow its progression, making diagnosis crucial for optimal care. Current screening guidelines recommend measuring blood creatinine -- which is used to estimate glomerular filtration rate (eGFR), -- and the urine albumin to creatinine ratio, an indicator of renal endothelial inflammation. Adherence to these screening recommendations can enhance patient care and reduce healthcare costs [1], [2].

A recent study by Alfego and colleagues revealed that up to 80 % of *at-risk* individuals have not received proper CKD screening [3]. This includes a sizable 10.4 % who missed out on creatinine-based eGFR measurements, despite the inclusion of creatinine in both basic and complete metabolic panels. Several factors contribute to this underscreening for CKD. First, there is a general lack of public awareness about the prevalence of CKD. Second, many people are unaware that there are treatment options available if CKD is diagnosed. Third, many people find laboratory services inaccessible due to geographical location, socioeconomic factors, or lack of healthcare coverage [4].

The earlier CKD is detected, the easier it is to slow its progression to end-stage renal disease, a dialysis-dependent condition that may necessitate a kidney transplant. People with lower socioeconomic status tend to have a higher prevalence of CKD, limited access to treatments, and overall poorer outcomes [1], [3], [5]. Therefore, it's imperative to develop accessible screening programs for those living under these constraints. Multiple studies have highlighted the success of home testing for albuminuria screening. One such study reported similar adherence rates across different income categories and population densities, and higher adherence among racially minoritized participants [6], [7].

Creatinine is traditionally measured in serum or plasma using high-throughput chemistry analyzers, which requires the collection of a venous sample. Point-of-care devices are available and have shown varying degrees of success in novel screening programs [8], [9], [10]. The use of capillary blood spotted onto filter paper for dried blood spot (DBS) analysis could enhance access to CKD screening. This method allows for remote collection and postal delivery of the self-collected samples [11], [12]. Since the extraction from DBS yields a minimal sample volume, measuring creatinine concentration requires an alternative method. In this study, we developed a streamlined LC-MSMS method capable of accurately and precisely quantifying creatinine from DBS samples.

## Methods

### Samples

Venous and capillary samples with creatinine concentrations within the reference interval were collected in various matrices (DBS, serum, and lithium heparin plasma (LiHep)) from consenting participants at LetsGetChecked Laboratories (Monrovia, CA) under an IRB approved protocol (ISDFS2018). Additionally, residual de-identified LiHep whole blood samples with elevated creatinine concentrations were received from the University of North Carolina at Chapel Hill and Penn Medicine Lancaster General Hospital.

DBS samples were prepared either by “free-fall” from the finger or by pipetting 40 Âµl of whole blood onto a Whatman 903 card. The free-fall samples were collected using one of two approaches, a controlled approach, and an unsupervised approach. For the controlled approach, a trained phlebotomist used a lancet to puncture the participants’ finger and applied pressure to the finger to release 1–2 drops of blood onto the pre-defined circle. For the unsupervised collections participants were provided collection instructions (supplemental Fig. S1) and the necessary supplies to collect a DBS without additional consultation or assistance from phlebotomy. All DBS samples were evaluated regardless of their visible appearance, but in production our procedure specifies to reject samples that are underfilled, overfilled, or spot outside of the pre-defined margin. These rejection criteria are also listed in our collection instructions.

For the seasonal stability experiments, samples with concentrations within the normal range of creatinine (<2 mg/dL) were collected free-fall (n = 110 using the controlled free fall collection); all samples with higher concentrations (>2 mg/dL; n = 30) were manually pipette using 40uL of residual LiHep whole blood. For the method comparison, the same collection strategies were used, but an additional set of DBS samples (n = 30) were collected using the unsupervised approach. All DBS cards were allowed to dry for at least 24 h before analysis.

### Preparation of standards

A 7-point matrix-matched calibration curve (0.2, 0.4, 0.8, 1.0, 5.0, 10.0 and 20.0 mg/dL) was made by taking a baseline whole blood sample (LiHep) and manipulating the specimen to yield seven defined concentrations, as follows. An aliquot of the neat sample was used as a mid-concentration (0.8 mg/dL). For calibration points greater than 0.8 mg/dL, creatinine certified reference material was added to increase the concentration (Cerilliant, Round Rock, TX). For calibration points below 0.8 mg/dL, red blood cells were diluted with an isotonic solution (Alsever’s Solution, Sigma-Aldrich, St. Louis, MO). For all points, a portion of the resulting whole blood calibrator was centrifuged and the plasma was used to determine the creatinine concentration using the Roche Cobas c502 enzymatic assay. After verification that the concentrations were near that expected, 40 uL of each matrix matched whole blood calibration point was spotted.

Ultimately, the expected target concentrations of the resulting DBS calibrators were defined using two challenge sets. Both challenge sets consisted of 40 paired DBS/plasma patient samples with expected concentrations derived from the Roche Enzymatic assay. The first set of 40 DBS samples were used to define the calibration curve values (the DBS calibrators described above were run as unknowns). The assigned concentrations for the DBS calibrators were used as the target value for future calibration curves. The second set of 40 samples challenged the defined calibrator values to ensure creatinine results were harmonized with the Roche enzymatic assay. The peer group mean/SD/CV of the Roche assay comparator assay is 0.86 mg/dL/0.04/4.8 % for level 1, 2.0 mg/dL/0.051/2.6 % for level 2, and 6.7 mg/dL /0.16/2.3 % for level 3.

### Preparation of quality controls

Matrix-matched quality controls were created using four LiHep whole blood samples chosen to span the AMR (0.3–20.0 mg/dL). Target values were defined by taking an aliquot of whole blood from each sample, centrifuging, and measuring the plasma creatinine using the Roche enzymatic assay (resulting concentrations 0.74, 0.95, 5.98, 14.72 mg/dL). The whole blood was spotted onto Whatman 903 cards to make four DBS QC levels. After spotting, DBS QC cards were stored at room temperature in a desiccant box.

### Extraction procedure

A 6 mm punch was used for analysis of calibrators, quality control, and patient samples. Once punched, 150 uL of water (Sigma-Aldrich, St. Louis, MO) with 0.06 mg/dL of creatinine-D3 internal standard (Cerilliant, Round Rock, TX) was added and the samples were incubated (15 min, 60℃) and then shaken (5 min, 1200 RPM). After shaking, 684 uL of acetonitrile (Sigma-Aldrich, St. Louis, MO) was added and the mixture was shaken (5 min,1200 RPM) and centrifuged (10 min, 5000 RPM, 5℃). The resulting supernatant was diluted by transferring 20uL into 384 uL of an Acetonitrile: Water (80:20) mixture. The dilution was shaken (30 sec, 1200 RPM) before injecting 10uL into the LC-MS/MS.

The extraction method detailed above was automated using a Microlab Star liquid handler (Hamilton Company, NV). First, the DBS cards were manually scanned and punched into a 96 deep-well plate (Axygen, Corning, AZ). Following, the liquid handler added the internal standard solution to each well. The plate was manually removed and placed on a heated shaker (Bioshake iQ, QINSTRUMENTS, Germany). Once complete, the plate was manually returned to the liquid handler and acetonitrile was added before removing the plate and placing it back onto the shaker (ambient temperature). Once the protein precipitation was completed, the plate was centrifuged with cooling temperatures (Allegra X15R, Beckman Coulter, CA). Finally, the liquid handler removed an aliquot from the sample plate and transferred it to the analysis plate, which was shaken at room temperature before injecting the sample onto the column.

### Analyte separation and instrument acquisition parameters

Chromatographic separation of creatinine and the internal standard was achieved using a Hypersil™ Silica, 100 X 2.1 column with a 5 µ particle size on an Agilent 1260 infinity system (Santa Clara, CA). The mobile phase used for separation consisted of 10 mM ammonium formate (Sigma-Aldrich, St. Louis, MO) in 80 % acetonitrile and 20 % water (Sigma-Aldrich, St. Louis, MO). Creatinine was eluted using an isocratic mobile phase over 2 min with a constant flow rate of 1 mL/min; creatinine and its internal standard eluted at 0.4 min. Creatinine quantification was performed using an API 4000 triple quadrupole mass analyzer (SCIEX, Redwood City, CA) with an ESI source operated in positive ionization with multiple reaction monitoring (MRM). Analytical parameters were as follows; ion spray voltage: 4500 V; source temperature: 550 ℃; curtain gas: 25; collision gas: Medium; Ion Source Gas 1: 65; Ion Source Gas 2: 40; dwell time: 225 ms; declustering potential: 61 V; collision energy: 17 V; collision exit potential: 16 V. Ion transitions monitored for creatinine and its internal standard were (114.0 → 86.8 *m*/*z*, 114.0 → 44.5 *m*/*z*) and (117.0 → 89.9, 117.0 → 47.5 *m*/*z*), respectively.

Ion ratio acceptability was defined after calculating the ion ratios over five sets of calibrators run in triplicate on five separate days. The relative intensity of the qualifier was 70 % of the quantifier, which according to CLSI Document C50-A allows a maximum permitted tolerance of ± 20 %. Thus, the ion ratio for the QC and patient samples must be within ± 20 % of the average ion ratio of the calibrators within the specific run to be considered acceptable.

### Precision and accuracy

Inter and intra-day precision were evaluated by running each DBS QC level in replicates of five for ten consecutive days (expected concentrations 0.74, 0.95, 5.98, and 14.72 mg/dL). Observed means, standard deviations (SDs) and coefficient of variation (%CV) were calculated for all DBS QC levels.

To evaluate accuracy, the four DBS QC materials were run in replicates of five to attain the observed values. The expected concentration was established by running all four control levels (plasma aliquot) on the Roche Cobas 502 enzymatic assay., The difference between observed and theoretical analyte concentration divided by the theoretical concentrations multiplied by 100 (% deviation), was calculated.

### Linearity

Linearity was confirmed by running five levels of DBS calibration standards for five consecutive days. The standard curve was calculated using the ratio of the peak area of creatinine to creatinine-IS using a 1/x weighted linear regression running through zero (y = 0). Data was evaluated using the EP Evaluator CLSI EP6 Linearity module.

### Interference assessment

To ensure that creatinine was successfully resolved from creatinine, a whole blood patient sample was divided into two aliquots. Exogenous creatine was added to one of the aliquots to a final concentration of 5 mg/dL. The chromatogram was visually inspected for peak interference and the quantitative results were compared.

Triglyceride interference was assessed by comparing creatinine concentration in a paired whole blood/plasma sample with a normal concentration of creatinine, but high concentration of triglycerides (2178 mg/dL). The DBS sample was extracted in triplicate; all extractions and the plasma sample were run three times. Plasma results from the enzymatic assay were compared to the DBS results and % recovery was calculated.

Bilirubin and M−protein interferences were assessed using residual plasma samples with high bilirubin (n = 6, 5.4–16.5 mg/dL total bilirubin and 2.3->10 mg/dL direct bilirubin) or immunoglobulins (n = 1 IgG M−protein (3388 g/L); n = 2 IgA M−protein (52,981 and 8,023 g/L); n = 1 IgM M−protein (3716 g/L), respectively. The residual plasma was mixed 1:1 with washed red blood cells and inverted to mix. The subsequent samples were spotted onto Whatman DBS in triplicate. The remaining whole blood was spun and the plasma supernatant was used to determine the expected creatinine concentration.

For isobaric interference, a 30 mg/dL creatinine sample in water was extracted and intensity at the creatinine-D3 mass:charge expected peak location was monitored. Conversely, we used stock IS solution (10 mg/dL creatinine-D3 in 50/50 water/methanol) to see if a change in intensity at the creatinine mass:charge expected peak location would be observed.

### Spot volume assessment

A range of LiHep whole blood volumes from the same sample were spotted onto DBS cards (20, 40, 60, 80, and 100 uL). These volumes remained within the pre-defined boundaries of the Whatman card. Each resulting spot was run in replicates of five and compared to the target value set by the enzymatic Roche creatinine assay.

### Hematocrit assessment

LiHep whole blood samples (n = 6 healthy donors) were used to see how variations in hematocrit would influence results. Each sample was manipulated to create three different hematocrit concentrations, as follows. First, the red cells were gravity separated from the plasma at ambient temperature. To make a 30 %, 40 %, and 55 % hematocrit, 140 uL, 120 uL, and 90 uL of the resulting plasma supernatant was mixed with 60 uL, 80 uL, and 110 uL of the red cell sediment, respectively. The resulting samples were spotted and allowed to dry before analysis. Each sample was extracted and evaluated in triplicate and the results were compared to the Roche enzymatic creatinine from the neat plasma.

### Stability

Room temperature stability was evaluated using venous whole blood collected without preservatives/anticoagulants (n = 2 healthy volunteers with normal creatinine concentrations). The whole blood was spotted immediately after collection to avoid clotting. The resulting DBS samples were tested in replicates of five for 10 consecutive days.

Seasonal temperature stability was assessed by comparing the concentration of creatinine in DBS before and after exposure to extreme heat and winter challenges. For these experiments, samples with concentrations within the normal range of creatinine (<2 mg/dL) were collected free-fall (n = 110 using the controlled free-fall collection); all samples with higher concentrations (>2 mg/dL; n = 30) were manually pipetted using 40uL of residual LiHep whole blood. A Binder chamber (KB E4) was used to create winter (cycling between −10° and 22 °C) and summer (cycling between 22° and 40 °C) conditions using general FDA-recommended shipping protocols.

### Matrix effects

Matrix effects were evaluated as previously described by Matuszewski et al [13]. Ion suppression/enhancement for creatinine was assessed by creating three different samples, each containing 150 uL of water with 0.06 mg/dL of creatinine-D3 internal standard. The first sample was composed of water spiked with 5 mg/dL of creatinine and evaluated “wet” (Sample 1). The second sample was composed from LiHep whole blood spiked with 5 mg/dL of creatinine and then spotted onto a DBS card (Sample 2). The third sample was composed using the same LiHep whole blood sample used for Sample 2, but spotted without addition of exogenous creatinine to screen for endogenous creatinine concentration (Sample 3). Concentration ratios for analyte and internal standard were used to determine overall matrix effects (comparison of Sample 2 to Sample 1), extraction efficiency (comparison of Sample 2 to Sample 3) and process efficiency (comparison of Sample 3 to Sample 1).

Additionally, the CV of the internal standard signal for all calibration (0.2–20 mg/dL), QC (4 levels of patient-based, matrix matched QC), and patient samples (n = 11) for a typical run was plot to determine if ion suppression was observed.

### Method comparison

Paired venous plasma/DBS samples (for DBS collection n = 113 controlled free-fall; n = 30 unobserved free-fall; n = 30 manually pipette) with concentrations spanning the AMR were compared between the LC-MSMS (DBS) and the Roche Cobas 502 enzymatic (plasma CREA2) creatinine assays.

In addition, a subset of these samples (n = 40; DBS collected using controlled free-fall) were compared between the LC-MSMS DBS assay, Beckman coulter AU5800 (OSR6678; Jaffe), Siemens Atellica CH 920 (eCre2 assay; enzymatic)and Abbot i-STAT (CREA; enzymatic). All measurements were performed in CAP accredited CLIA certified laboratories and had an SDI <=1.6 for all samples in the most recent CAP proficiency testing challenge.

### Data analysis

Results were derived from calibration concentrations by calculating the ratio between the peak areas of creatinine and the internal standard. The subsequent ratios were plotted, and linear regression was applied with a 1/x weighting. The derived equation was then used to calculate the results of unknown and QC samples. Analyst 1.7 software (SCIEX, MA) was used to facilitate this analysis. Calculations for the validation assessment, which included precision, accuracy, stability, and matrix effects, were performed using EP Evaluator 12.0.0.11 software (Data Innovations, VT), Google Sheets (Alphabet Inc., CA), and Prism 9.0 (GraphPad Software, MA). The error budget for all experiments was 0.2 mg/dL or 15 %, which is more stringent than what is currently recommended by CAP and CLIA [14], [15].

## Results

### Chromatographic separation

**Fig. 1:**
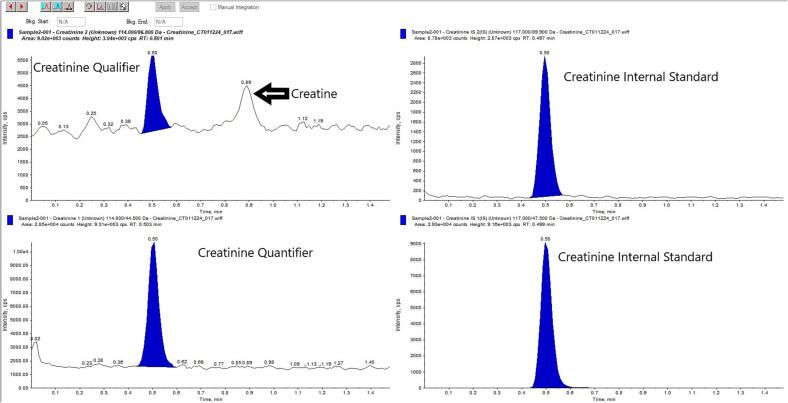
Chromatogram of a typical patient sample extracted and resolved using our DBS creatinine method. This whole blood sample was spiked with exogenous creatine before spotting to illustrate the resolution of this creatinine from its precursor compound.

Creatinine was successfully resolved from the void volume and creatine (Fig. 1). The void volume eluted at 0.24 min (0.24 mL); the creatinine and internal standard eluted at 0.4 min. Creatine eluted at 0.83 min.

### Precision and accuracy

Table 1 lists the intra- and inter-assay precision and accuracy of DBS creatinine at low-1, low-2, mid, and high QC concentrations. Across ten independent runs, total precision ranged from 5.2 % to 8.1 %; total accuracy ranged from 98.8 to 102.7 % relative to the Roche targets.

**Table 1:** Total precision and accuracy for creatinine in DBS by LC-MS/MS.

Creatinine QC	Roche Target (mg/dL)	Mean	SD	%CV	%Deviation	%Accuracy
Low-1	0.74	0.76	0.039	5.2	−2.7	102.7
Low-2	0.95	0.94	0.06	6.6	1.05	99.0
Mid	5.98	5.91	0.44	7.7	1.17	98.8
High	14.72	14.6	1.15	8.1	0.82	99.2

### Linearity

The assay was linear from 0.3 to 20.0 mg/dL. A representative 7-point DBS calibration curve fits to the equation y = 1.02x − 0.11; r^2^ = 0.9964.

### Interference assessment

High triglyceride concentration (2178 mg/dL) did not interfere with creatinine measurements on the DBS LC-MSMS method. The plasma creatinine concentration measured by the enzymatic Roche assay was 0.77 mg/dL, the DBS creatinine concentration by LC-MSMS was 0.75 ± 0.02 mg/dL; the difference between the two measurements was 2.6 %.

Bilirubin did not interfere with creatinine concentration. The average bias between the Roche and the DBS assay creatinine concentration across the six samples was 0.12 mg/dL or 4.6 %.

IgG and IgA did not interfere with results (average bias from the three samples was −0.07 or −9.3 %), but IgM falsely elevated the creatinine result by 0.34 mg/dL (40 %). Visually, the IgM sample was different from the others (Supplemental Fig. S2), as the blood did not properly absorb into the Whatman paper.

No isobaric interference was observed (data not shown).

### Effect of spot volume on whole blood creatinine results

**Fig. 2:**
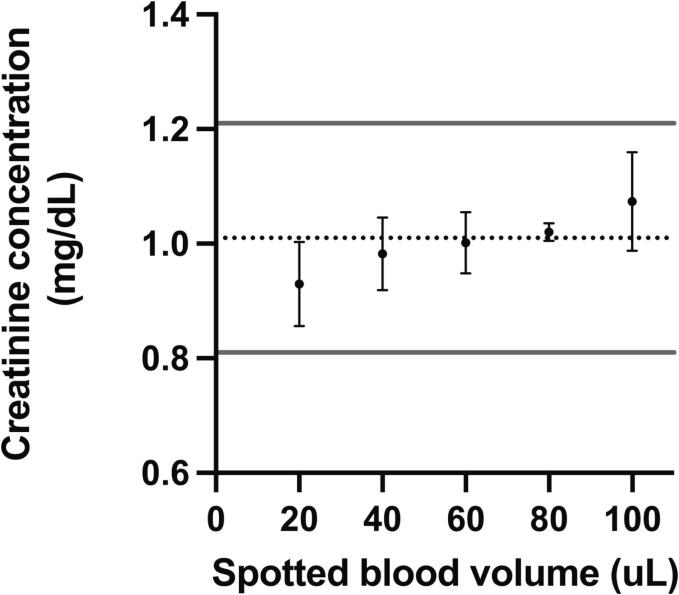
Assessment of spotting volume on creatinine concentration. Each point reflects the mean and SD of three extractions from a DBS spotted with the indicated volume. Solid grey line is the expected concentration based on the Roche enzymatic assay. Dotted grey lines are the +/-0.2 mg/dL error limits.

A positive bias in DBS creatinine measurement was observed with increasing spotted volume (Fig. 2). The mean result increased from 0.93 ± 0.07 to 1.07 ± 0.09 mg/dL as the spotted volume increased from 20 uL to 100 uL. This increase stayed within the allowable error of the target values established by the Roche enzymatic assay (1.01 ± 0.2 mg/dL).

### Effect of hematocrit on whole blood creatinine results

**Fig. 3:**
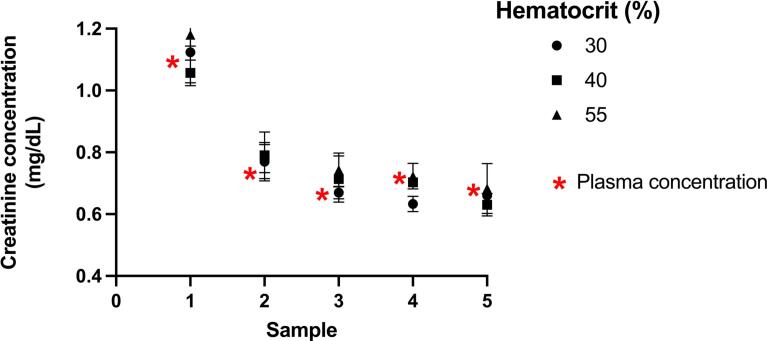
Assessment of hematocrit on creatinine concentrations across six different participant samples. The red asterisk indicates the expected concentration based on the paired plasma sample measured on an enzymatic creatinine assay. (For interpretation of the references to colour in this figure legend, the reader is referred to the web version of this article.)

Differences in hematocrit did not significantly impact results (Fig. 3). The delta for 30 % hematocrit compared to 55 % was 0.06, 0.01, 0.07, 0.09, and 0.02 mg/dL for Samples 1–5, respectively. Relative to the plasma concentration measured by the Roche enzymatic assay results from any level of hematocrit met the accuracy target for all samples.

### Stability challenges

**Fig. 4:**
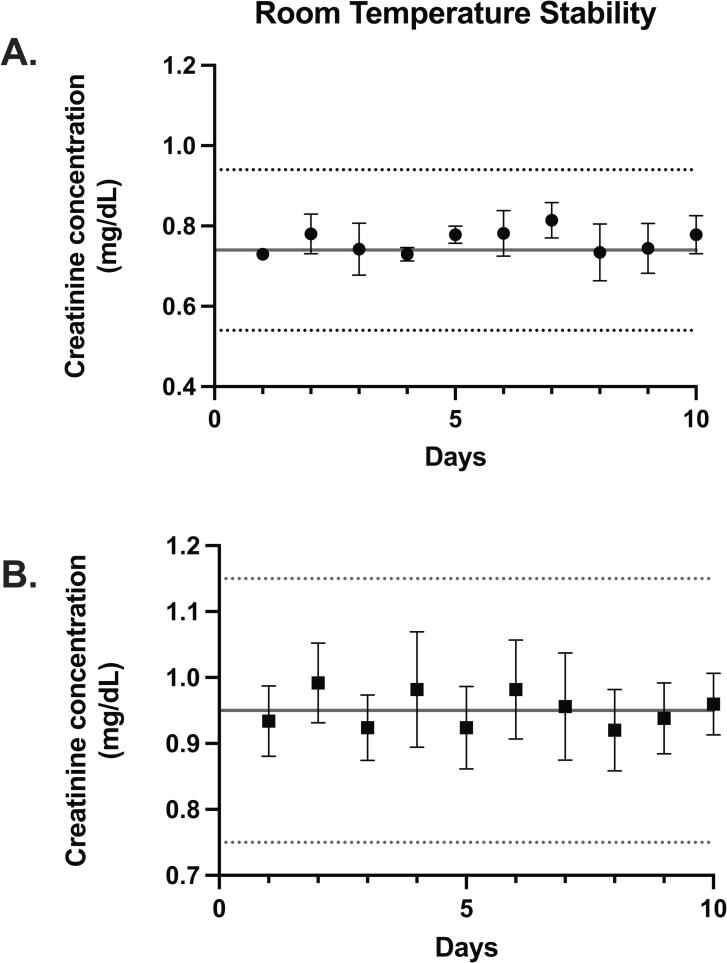
Room temperature stability of two sets of DBS samples collected without preservative and run in triplicate for 10 days. Each point reflects the mean and SD of three extractions.

**Fig. 5:**
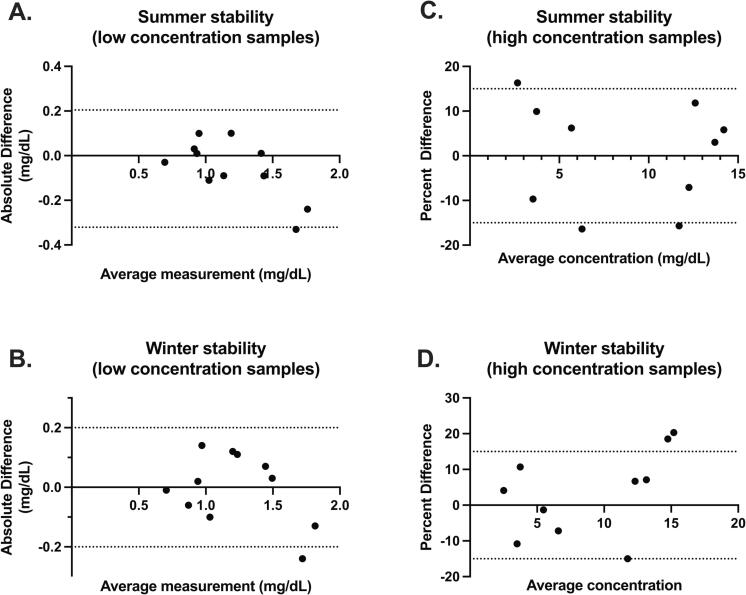
DBS samples collected free-fall directly from the finger (n = 11 participants collecting two cards each; creatinine concentrations < 2 mg/dL; panels A & B) or spot using residual LiHep whole blood samples (n = 10; creatinine concentrations > 2 mg/dL; panels C & D) were challenged with extreme summer (A & C) or winter (B & D) conditions. Results were compared to the expected concentration based on the Roche enzymatic assay measured on unchallenged venous plasma.

Stability challenges from room and extreme temperature conditions are illustrated in Fig. 4, Fig. 5. After storage at room temperature, the bias between the baseline and 10-day creatinine concentrations was −2.87 % or −0.02 mg/dL (Fig. 4A) and −0.26 % or −0.002 mg/dL (Fig. 4B) −0.01. The average standard deviation between the 100 replicates over 10 days was ± 0.06 mg/dL with an average coefficient of variation of 6.92 %.

For the summer stability challenge the bias between the average baseline samples and the average post-shipment samples was −0.063 mg/dL or −1.5 % (Fig. 5), which was within the defined error limit of 0.2 mg/dL or 15 %.

For the winter stability challenge the bias between the average baseline samples and the average post-shipment samples was 0.218 mg/dL or 4.6 % (Fig. 5), which was within the defined error limit of 0.2 mg/dL or 15 %.

### Matrix effects

Mean percentage matrix effects were 105.4 %. Extraction of creatinine from spotted whole blood showed decreased, although reproducible recovery efficiency at 79.9 % across the measuring range of the assay. Processing efficiency for creatinine was 103.1 %. Table 2 summarizes creatinine matrix effects, recovery efficiency, and processing efficiency.

**Table 2:** Summary of matrix effects, recovery, and processing efficiency.

Creatinine	% Matrix Effects	%Recovery Efficiency	%Processing Efficiency
Mid 5 (mg/dL)	105.4	79.85	103.07
(SD/%CV)	0.30/4.62	0.60/9.31	0.49/7.615

The CV of the internal standard across a representative run of calibration (n = 7), QC (n = 4) and patient samples (n = 11) was < 5 % (Supplemental Fig. S3).

### Plasma, whole blood, and DBS method comparison

**Fig. 6:**
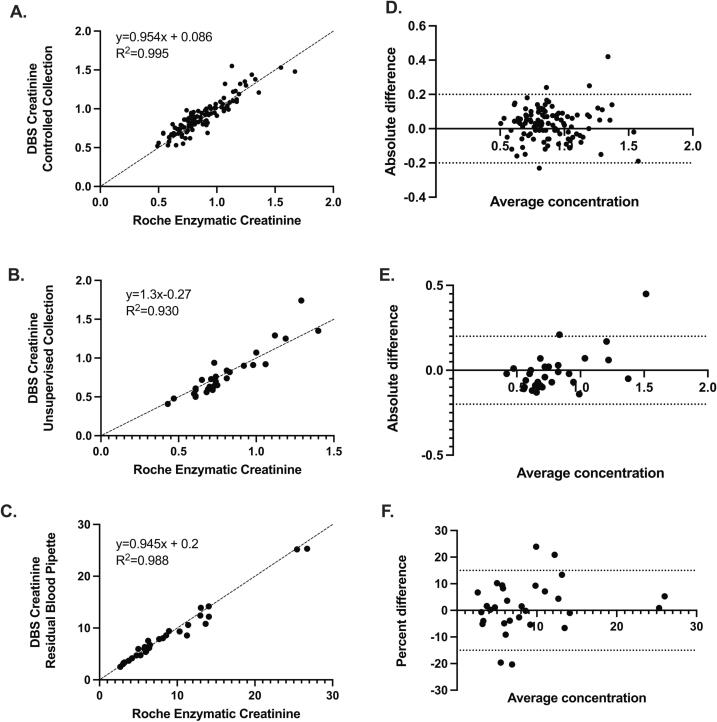
Method comparison between the Roche enzymatic assay (venous plasma) and DBS LC-MSMS method for samples < 2 mg/dL spot as DBS using a free-fall controlled collection (Fig. 6A; n = 113), samples < 2 mg/dL spot as DBS using a free-fall unsupervised collection (Fig. 6B; n = 30), and samples >=2mg/dL spot as DBS with pipette and residual LiHep whole blood (Fig. 6C; n = 30). Dotted lines show error limits of total allowable error 0.2 mg/dL (Fig. 6D & E) or 15 % (Fig. 6F). Line of identity illustrated with dashes (Fig. 6A-C). All creatinine concentrations in mg/dL.

As intended by the calibration procedure, the comparison between the enzymatic Roche assay (plasma) and the LC-MSMS (DBS) (n = 173) showed an average bias of −0.04 mg/dL and −1.4 %. When dividing the data between low (0.0–2.0 mg/dL; n = 113) and high (2.0–25 mg/dL; n = 30) concentrations, the average bias was 0.03 mg/dL or 3.4 % (Fig. 6**A**) and −0.28 mg/dL or −3.2 % (Fig. 6**C**), respectively. For the unobserved collection (Fig. 6**A**), overall comparison was similar to the controlled collection (Fig. 6**B**). The unobserved DBS creatinine results that exceeded the error budget of 0.2 mg/dL (n = 2; results from card:venous 1.74:1.29 mg/dL and 0.94:0.73 mg/dL) were visibly spot with excess blood (Supplemental Fig. S2) and, therefore, would have been rejected per our general acceptance criteria.

**Fig. 7:**
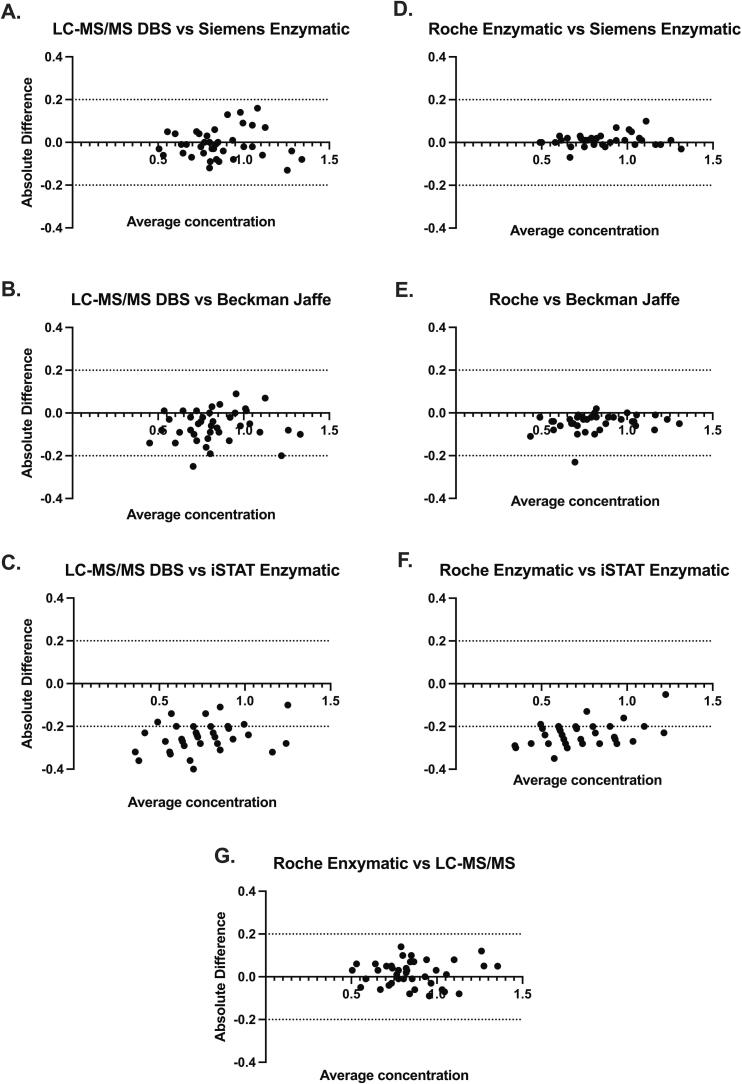
Bland-Altman plots (n = 40 samples) illustrating inter-instrument variability in creatinine concentration between theLC-MSMS creatinine method (spot as DBS using free-fall controlled collection; Fig. 7A-C,G) or the Roche enzymatic assay (venous plasma; Figures D-G) to alternative instrument/assay pairs. Creatinine assay comparators are as follows: **A and D** Siemens enzymatic; B and E. Beckman Jaffe; C and F. iSTAT enzymatic. Panel G is the identical 40 samples compared between the Roche enzymatic and LC-MSMS assays. Dotted lines show error limits 0.2 mg/dL.

A subset of these samples (n = 40) was compared across several instrument/assays. Comparison of these subset of samples between the Roche enzymatic assay (plasma) and the LC-MSMS (DBS) had an average bias of 0.02 mg/dL or 2.6 % (Fig. 7**G**).

Comparison of these 40 samples between the enzymatic Roche assay (plasma) or the LC-MSMS assay (DBS) and the Abbott iSTAT point-of-care device (whole blood) showed a negative average bias of −0.23 mg/dL or −27.2 % (Fig. 7**F**) and −0.25 mg/dL or −28.8 % (Fig. 7**C)**, respectively.

Comparison of the 40 samples between the enzymatic Roche assay (plasma) or the LC-MSMS assay (DBS) and the Beckman Jaffe assay (plasma) showed a slight negative bias of −0.034 mg/dL or −4.1 % (Fig. 7**E**) and −0.056 mg/dL or −6.5 % (Fig. 7**B)**, respectively.

Comparison of the 40 samples between the enzymatic Roche assay (plasma) or the LC-MSMS assay (DBS) and Siemens enzymatic assays (plasma) showed an average bias of 0.01 mg/dL or 1.1 % (Fig. 7**D**) and −0.01 mg/dL or −0.8 % (Fig. 7**A)**, respectively.

## Discussion

Here, we developed an LC-MSMS method to measure creatinine from DBS. Our method is similar to previously published methods that use LC-MSMS to measure serum/plasma creatinine [16] or methods for which the focus was on therapeutic drug monitoring [17] and not CKD screening. We demonstrate that our method compares to two enzymatic creatinine assays with higher accuracy than a Jaffe assay (Beckman Coulter, AU series) or a point-of-care device (Abbott, iSTAT). Further, we show that several pre-analytical variables such as exposure to extreme temperatures, differing applied blood volumes, or changes in hematocrit do not influence the accuracy or precision of results. Ultimately, we strive to utilize this method to increase screening access in underserved and remote communities, and to support awareness campaigns related to CKD screening.

Similar to other methods, using matrix-matched calibration was crucial in ensuring the accuracy of our creatinine results [18]. DBS are self-collected, making it impossible to ensure the same amount of blood is applied to the card for each collection. Similarly, hematocrit, or the red cell volume of a sample, can influence plasma volume by up to 14 % between non-anemic individuals (based on the sex-specific reference intervals), thereby potentially altering result accuracy. We hypothesize that because our calibration values were derived from dozens of different individuals who applied blood directly from the finger onto the filter paper, this inter-individual variability was accounted for.

Positive interference was observed in a sample with excess IgM. Additionally, when the comparison to the enzymatic venous concentration was challenged with an unobserved DBS collection, 2 of 30 samples (6.7 %) had an unacceptably elevated bias. For both types of interference, laboratory rejection criteria would have mitigated the reporting of these results. For the IgM sample, the blood did not fully penetrate the filter paper (as can be seen in the Supplemental Figure middle photos); for the unobserved collection, the excess application of blood bridged the pre-defined margins (Supplemental Fig. S2 bottom photos). Importantly, these pre-analytical factors resulted in falsely elevated results, which is preferable for a screening method.

Our method has some limitations. We were unable to obtain free-fall capillary samples collected directly from the finger from people with known CKD, and, therefore, used residual EDTA or LiHep whole blood pipetted onto the filter paper as a surrogate. Additionally, our method was based on a simple harmonization between the LC-MSMS and the Roche enzymatic assay. Thus, our assay is only indirectly traceable to an IDMS reference measurement procedure. Lastly, although acceptable, our precision at the high end was less robust and our upper limit of linearity was below most clinical creatinine methods.

Accurate and precise measurements of significantly elevated creatinine concentrations are most important for people in acute or critical care settings, as the delta creatinine in these patients is closely monitored and a 10–15 % shift could be actionable. However, for screening, a single eGFR measurement < 60 mL/min/1.73 m2 is not diagnostic of CKD or appropriate for staging. It indicates that referral to nephrology and/or follow-up testing is required. eGFR values can be sporadically low depending on diet, water balance, medications, and exercise regimen. As such, the eGFR range between 60 and 90 mL/min/1.73 m2 provides a buffer as an indeterminate range, during which creatinine should be reassessed in conjunction with a urine albumin to creatinine ratio. We do not expect people to trend eGFR or creatinine concentration with this method. Rather, this assay is meant to be used to improve access to CKD screening in at-risk populations.

The development and validation of this method is only the first step. For our method to be successful, it is imperative to design operational pathways that allow for scalability. Unlike wet samples that have many automation solutions to increase throughput, DBS are much more difficult to automate. Future directions will include system design to ensure a workflow that can accommodate large-scale screening. Additionally, we must continue to rigorously monitor the harmonization of results with an enzymatic assay by routinely using patient-based (matrix-matched) QC challenges and intermittent comparisons to ensure the stability of calibration. Ultimately, we hope to complete the necessary experimental processes for IDMS traceability. Lastly, we hope to monitor the clinical impact of offering this screening approach to people who would otherwise remain unscreened. CKD remains an underrecognized and underdiagnosed chronic condition, and early diagnosis is known to improve outcomes. Ensuring that there are multiple quality screening pathways for all people would ideally mitigate these disparities. In summary, we have developed and automated an LC-MSMS method to measure creatinine from DBS that is suitable for CKD screening.

## CRediT author statement

DNG and CT contributed to the supervision, conceptualization, methodology, software, resources, validation, formal analysis, data curation, and writing. ARN, RAM, SWC, and CCP supported the methodology, formal analysis, resources, data curation, and writing.

## Ethics statement

This study was conducted in accordance with the principles set forth in the Declaration of Helsinki and Good Clinical Practice (GCP) Guidelines. All samples for clinical validation were collected under an IRB approved protocol issued to LetsGetChecked Laboratories (Advarra IRB Pro00027040, IRB Organization Number: 0000635; IRB Registration Number: 00000971; Columbia, MD).

## Declaration of competing interests

The authors declare the following financial interests/personal relationships which may be considered as potential competing interests: at the time of writing, Dina Greene, Carlos Torres, Rogers Mudrow, and Anissa Naranjo were employed by LetsGetChecked. Additionally, Dina Greene receives editorial compensation from Elsevier and has been a scientific consultant for Abbott Diagnostics Point of Care.

## Declaration of competing interest

The authors declare that they have no known competing financial interests or personal relationships that could have appeared to influence the work reported in this paper.

## Figures and Tables

**Supplementary Fig. S1:**