# Supplementary material for: Development and validation of an LC-MSMS method to quantify creatinine from dried blood spots
Source: J Mass Spectrom Adv Clin Lab. 2024 Mar 6;32:50–9. doi: 10.1016/j.jmsacl.2024.03.001 (PMC10950697; doi:10.1016/j.jmsacl.2024.03.001)
Supplement: Supplementary data 1 [file mmc1.pdf]

LetsGetChecked Blood DBS and Urine Home Sample Collection IFU 111

SPECS AND INSTRUCTIONS

Colour Reference:
CMYK

Size:
108.5mm wide by 152.5mm high **folded**

Paper:
115gsm silk

Fold type:
6 page accordion

Font:
Proxima Nova
(Regular, Semibold and Bold)

Part Number:
QR-IFU-111-R0 Aug-2023

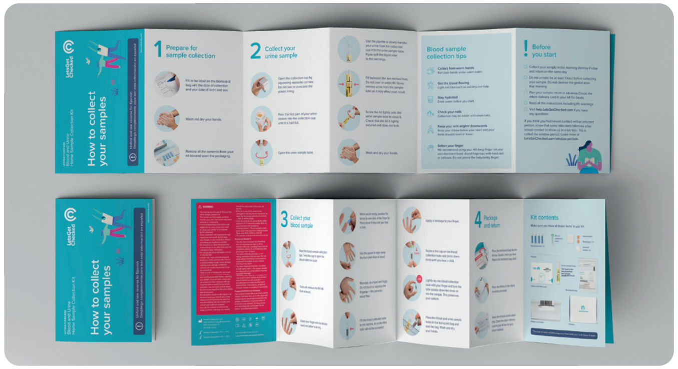

*Visual reference (showing the English side of the IFU) for how to fold down the sheet so the front page is the first one to see. Artwork and page count may vary.

LetsGet Checked

FOLD GUIDE

------ Fold

Accordion fold first

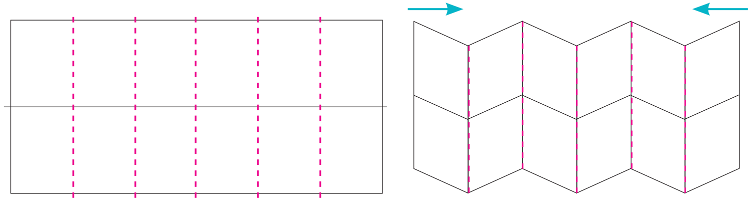

Then fold in a half with the text in English on the outside

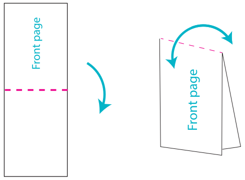

Outside – English version

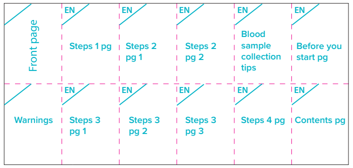

Inside – Spanish version

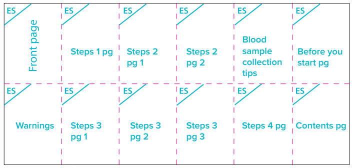

LETSGETCHECKED
Blood and Urine
Home Sample Collection Kit

How to collect your samples

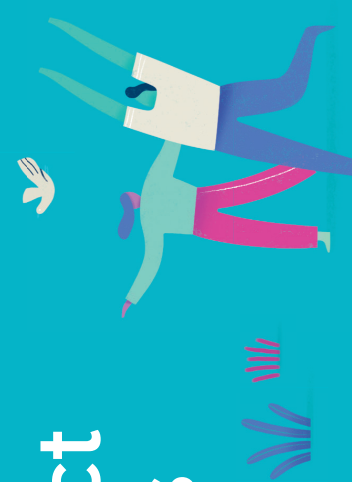

EN Unfold and see reverse for Spanish
ES Despliega y mira el dorso para leer esta información en español

QR-IFU-111-R0 Aug-2023

⚠ WARNING:

- You must be over the age of 18 to use this home sample collection kit.
- The samples and information provided must be your own. Test results may not be released to a third party.
- Failure to follow these instructions may impact the accuracy of your test result or cause your samples to be rejected by the laboratory.
- Some medication and supplements may impact your test result. Do not make any changes to your medication without consulting your healthcare provider.
- If you are collecting a sample for a test that requires fasting please consult with your healthcare provider, particularly if you have any medical conditions.
- Do not pierce or collect blood from the smallest finger or any other body part not indicated in these instructions.
- Do not use lancets on damaged, scarred, or infected skin.
- Lancets may cause some bruising and tenderness at the puncture site. There is a small possibility of pain, nerve damage, scarring, bone damage, or infection. Consult your healthcare provider if you are concerned.
- There is a risk of fainting with any blood sample. If you are concerned, consult your healthcare provider before collecting your sample. If you proceed, ensure you are seated and have someone with you. If you feel faint or unwell during sample collection, stop and seek medical advice.
- Use only the contents included in this single-use kit for collecting your samples. Check the expiry date before you use the kit.
- If the kit or any of the contents are damaged or missing, the kit should not be used. Do not tamper with the kit contents — Risk of serious injury.
- Used kit contents can generally be safely disposed of in household waste. Refer to your local laws and guidelines.
- Choking hazard – This kit contains small parts which may present a choking hazard. Keep away from children under 18.
- The samples must be returned in the kit box.

Do not use this kit if:

- You are at risk of fainting due to blood sample collection, including if you have a history of arrhythmia, fainting, or fear of blood.
- You have a bleeding disorder or are taking medication that increases the risk of bleeding.
- You have impaired lymphatic drainage in both upper limbs.
- You have a condition associated with poor healing, increased risk of infection, or skin ulceration — including but not limited to Raynaud's phenomenon, scleroderma, peripheral vascular disease, or peripheral neuropathy.

Purpose/Intended use of device:
The LetsGetChecked Blood and Urine Home Sample Collection Kit is an in vitro diagnostic medical device specifically intended for the primary containment and preservation of blood and urine samples, for the purpose of in vitro diagnostic examination by our partner accredited laboratories.

PrivaPath Diagnostics Ltd.,
Unit 1, Northern Cross Business Park,
North Road, Dublin 11,
D11 XT26, Ireland.

Storage temperature: 15°C — 25°C.
Transport temperature: 4°C — 25°C.

IVD

For explanation of symbols see **LetsGetChecked.com/symbol-glossary**

1 Prepare for sample collection

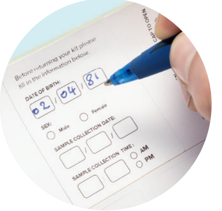

Fill in the label on the biohazard bag with the date of collection and your date of birth and sex.

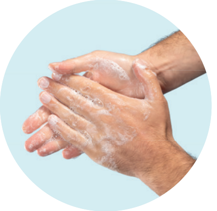

Wash and dry your hands.

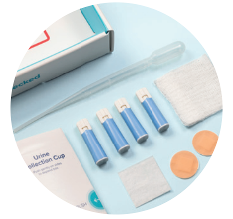

Remove all the contents from your kit box and open the packaging. The blood sample collection card must be kept on a clean dry surface. Do not touch the two circles.

2 Collect your urine sample

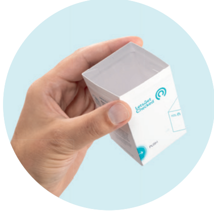

Open the collection cup by squeezing opposite corners. Do not tear or puncture the plastic lining.

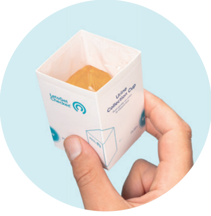

Pass a small amount of your urine into the toilet and then pass your urine into the collection cup until it is half full.

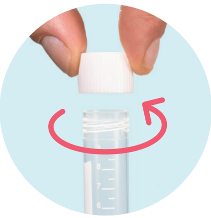

Open the urine sample tube.

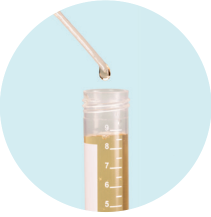

Use the pipette to slowly transfer your urine from the collection cup into the urine sample tube.

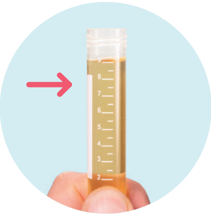

Slowly fill the tube until it is at least half full. Never remove urine from the sample tube as it may affect your result.

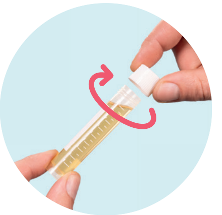

Screw the lid tightly onto the urine sample tube to close it. Check that the lid is tightly secured and does not leak.

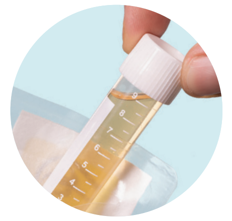

Place the urine sample tube in the urine sample return bag and seal the bag. Wash and dry your hands.

Blood sample collection tips

Collect from warm hands
Run your hands under warm water.

Get the blood flowing
Light exercise such as walking can help.

Stay hydrated
Drink water before you start.

Check your nails
Collection may be easier with short nails.

Keep your arm angled downwards
Keep your elbow below your heart and your hand at waist level or lower.

Select your finger
We recommend using your 4th (ring) finger on your non-dominant hand. Avoid fingertips with hard skin or calluses. Do not pierce the baby/pinky finger.

! Before you start

- ☐ Avoid protein-heavy meals, including red meat, protein shakes, and supplements, for 24 hours before collecting your samples
- ☐ Collect your samples in the morning Monday-Friday and return on the same day
- ☐ Plan your sample return in advance. Check the return delivery card in your kit for details
- ☐ Read all the instructions including the warnings
- ☐ Visit **help.LetsGetChecked.com** if you have any questions

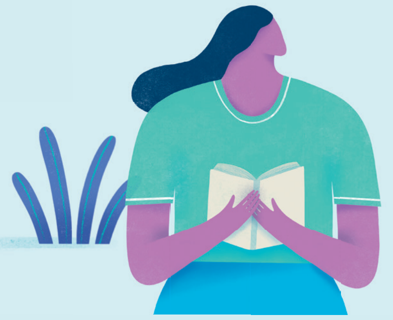

3 Collect your blood sample

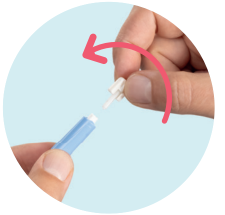

Read the blood sample collection tips. Twist off the white lancet cap. Do not pull it. Clean your finger with the alcohol swab and allow to air-dry.

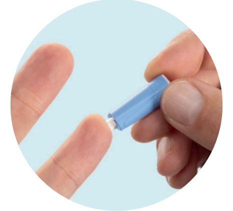

When you're ready, position the lancet to one side of the fingertip. Press down firmly until you hear a click.

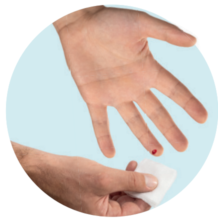

Use the gauze to wipe away the first small drop of blood.

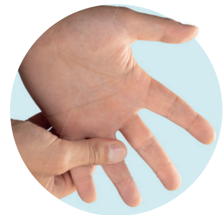

Massage your hand and finger. Do not touch or squeeze the fingertip — this prevents blood flow.

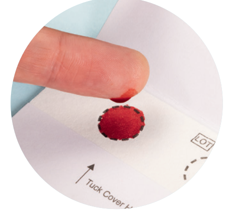

Drop blood onto a circle and fill it entirely as shown. The front and back of the circle should be completely red when complete. Repeat for the second circle. At least one circle must be completely filled.

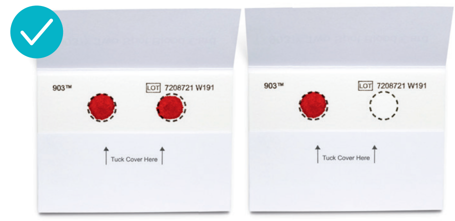

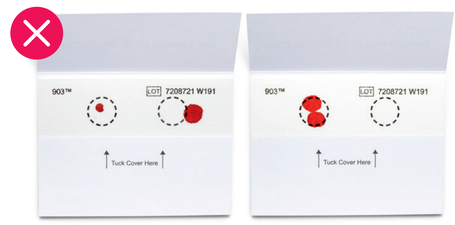

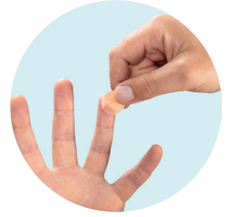

Apply a bandage to your finger.

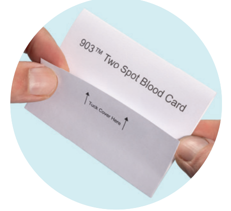

Fold down the cover of the blood sample collection card and gently tuck it under the flap (marked with arrows). Do not flatten or press down on the card.

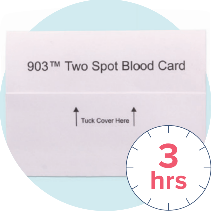

Allow the blood sample collection card to dry for 3 hours. Do not apply heat to assist drying.

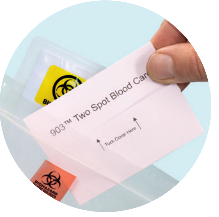

Place the urine sample return bag and the blood sample collection card into the biohazard bag and seal the bag.

4 Package and return

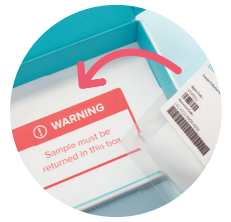

Place the biohazard bag into the kit box. Double check you have filled in the biohazard bag label.

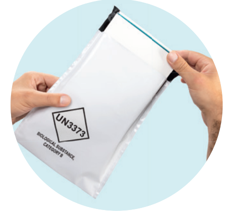

Place the kit box in the return envelope provided.

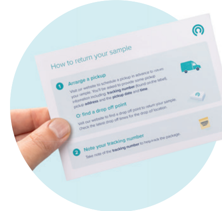

Send the kit back on the same day. Check the return delivery card in your kit box for your return method.

Kit contents

Make sure you have all these items in your kit:*

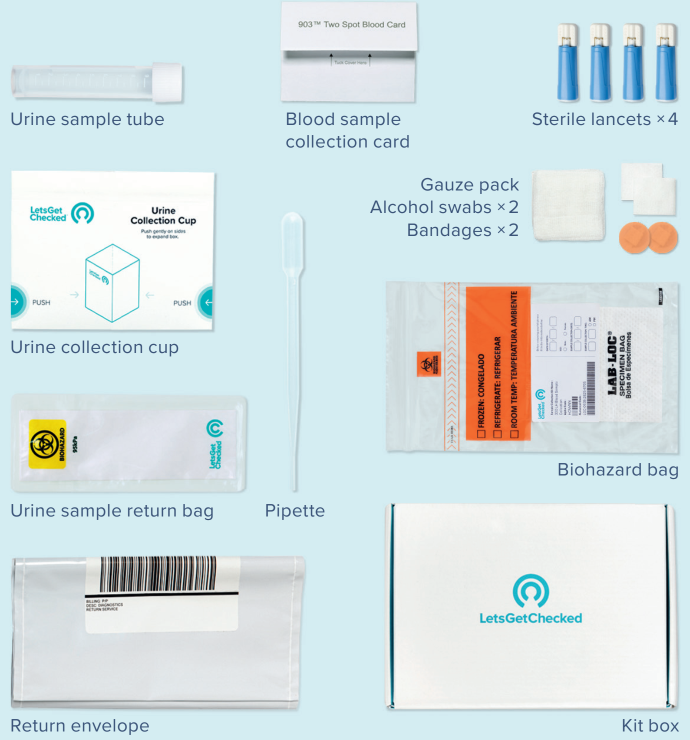

*The look or color of items may vary from what you see in these images.

LetsGet Checked

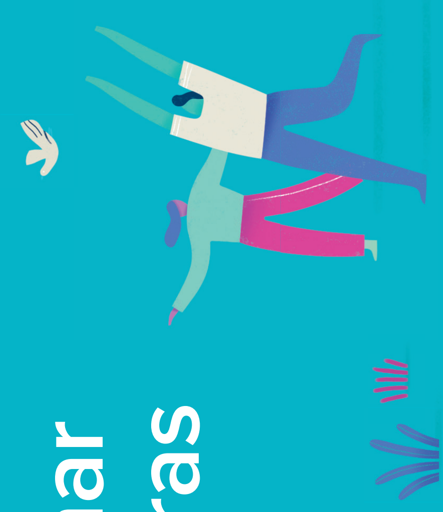

QR-IFU-111-R0 Aug-2023

LETSGETCHECKED
Kit casero de toma de muestras de sangre y orina

ES

Cómo tomar tus muestras

⚠ ADVERTENCIA:

- Debes ser mayor de 18 años para usar este kit casero de toma de muestras.
- Las muestras y la información que proporciones deben ser tuyas. Los resultados de las pruebas no se pueden divulgar a un tercero.
- No seguir estas instrucciones puede afectar la precisión del resultado de tu prueba o hacer que el laboratorio rechace las muestras.
- Algunos medicamentos y suplementos pueden afectar los resultados de la prueba. No modifiques tu medicación sin consultar con tu proveedor médico.
- Si estás tomando una muestra para una prueba que necesita ayuno, consulta con tu proveedor médico, especialmente si tienes alguna afección.
- No pinches ni tomes la muestra de sangre del dedo más pequeño (meñique) ni de ninguna otra parte del cuerpo que no se indique en estas instrucciones.
- No uses lancetas en piel dañada, con cicatrices o infectada.
- Las lancetas pueden causar hematomas y sensibilidad en el lugar de la punción. Existe una pequeña posibilidad de dolor, daño neural, cicatrización, daño óseo o infección. Consulta a tu proveedor de atención médica si te preocupa.
- Existe el riesgo de desmayos en cualquier toma de muestras de sangre. Si te preocupa, consulta con tu proveedor médico antes de tomar la muestra. Si tomas la muestra, asegúrate de estar sentado y de que alguien te acompañe. Si te sientes débil o mal durante la toma de la muestra, detente y busca atención médica.
- Usa solo el contenido que se incluye en este kit de un solo uso para tomar las muestras. Controla la fecha de vencimiento antes de usar el kit.
- Si el kit o alguno de los contenidos están dañados o faltan, no se debe usar el kit. No alteres el contenido del kit. Riesgo de lesiones graves.
- En general, los contenidos del kit usados pueden desecharse de manera segura con los residuos domésticos. Consulta las leyes y directrices locales.
- Riesgo de asfixia: este kit contiene partes pequeñas que pueden representar un riesgo de asfixia. Mantenlo fuera del alcance de menores de 18 años.
- Las muestras se deben enviar en la caja del kit.

No uses este kit si:

- Corres riesgo de desmayarte debido a la toma de muestras de sangre, incluso si tienes antecedentes de arritmia, desmayos o miedo a la sangre.
- Tienes un trastorno hemorrágico o estás tomando medicamentos que aumentan el riesgo de hemorragia.
- Tienes alteración del drenaje linfático en ambas extremidades superiores.
- Tienes una enfermedad relacionada con una mala cicatrización, un mayor riesgo de infección o ulceración cutánea, como el fenómeno de Raynaud, esclerodermia, enfermedad vascular periférica o neuropatía periférica, entre otras.

Finalidad/Uso previsto del dispositivo:

El kit casero de toma de muestras de sangre y orina LetsGetChecked es un producto sanitario de diagnóstico in vitro específicamente diseñado para la contención primaria y la preservación de muestras de sangre y orina, con fines de exámenes de diagnóstico in vitro a cargo de los laboratorios asociados.

PrivaPath Diagnostics Ltd.,
Unit 1, Northern Cross Business Park,
North Road, Dublin 11, D11 XT26, Ireland.

Temperatura de almacenamiento: 15 °C – 25 °C.

Temperatura de transporte: 4 °C – 25 °C.

IVD 18+

Para la explicación de los símbolos, consulta **LetsGetChecked.com/symbol-glossary**

1 Prepárate para tomar la muestra

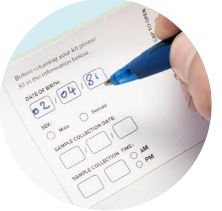

Registra la fecha de toma de la muestra, tu fecha de nacimiento y tu sexo en la etiqueta que se encuentra en la bolsa para residuos biológicos peligrosos. No toques los dos círculos.

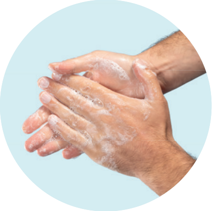

Lávate las manos y sécalas.

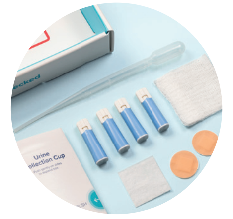

Retira todo el contenido de la caja de tu kit y abre el empaque. La tarjeta de toma de muestras de sangre debe conservarse sobre una superficie limpia y seca.

2 Toma tu muestra de orina

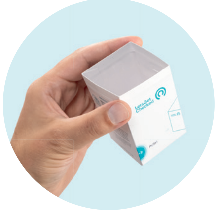

Abre el recipiente para tomar la muestra apretando las esquinas opuestas. No rasgues ni perfores el revestimiento de plástico.

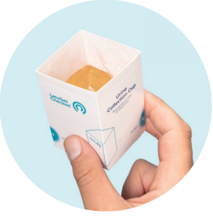

Orina una pequeña cantidad en el inodoro y luego dentro del recipiente para tomar la muestra, hasta llenarlo a la mitad.

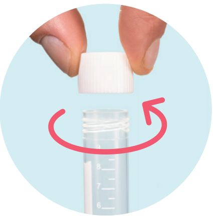

Abre el tubo de transporte de la muestra de orina.

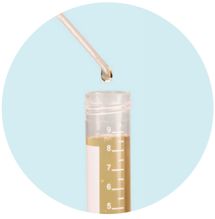

Con la pipeta, transfiere lentamente la orina del recipiente al tubo de transporte de la muestra de orina.

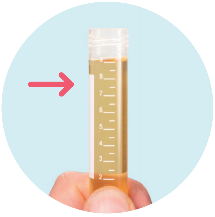

Llénalo lentamente hasta la mitad como mínimo. Nunca retires la orina del tubo para la muestra, ya que puede afectar su resultado.

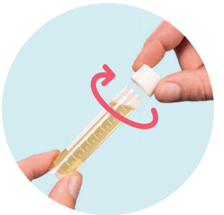

Enrosca con firmeza la tapa del tubo de transporte de la muestra de orina para cerrarlo. Comprueba que la tapa esté bien ajustada y que no tenga fugas.

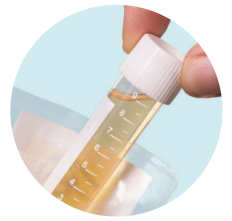

Coloca el tubo con la muestra de orina en la bolsa de envío de muestras de orina y sella la bolsa.

Consejos para la toma de muestras de sangre

Toma la muestra con las manos tibias
Coloca las manos debajo del agua tibia.

Toma la muestra cuando la sangre circule
Los ejercicios ligeros (como caminar) pueden ayudar.

Mantente hidratado
Bebe agua antes de comenzar.

Revisa tus uñas
La toma de la muestra puede ser más fácil con las uñas cortas.

Mantén el brazo inclinado hacia abajo
Mantén el codo por debajo del corazón y la mano a nivel de la cintura o más abajo.

Elige un dedo
Recomendamos usar el cuarto dedo (anular) de la mano no dominante. Evita las yemas de los dedos que tengan durezas o callos. No pinches el dedo meñique.

! Antes de empezar

- ☐ Evita las comidas ricas en proteínas, como la carne roja, los suplementos y los batidos de proteína, durante 24 horas antes de tomar las muestras
- ☐ Toma tus muestras por la mañana de lunes a viernes y envíalas el mismo día
- ☐ Planifica el envío de tu muestra con anticipación. Consulta la tarjeta de entrega de tu kit para más detalles
- ☐ Lee todas las instrucciones, incluidas las advertencias
- ☐ Visita **help.LetsGetChecked.com** si tienes alguna pregunta

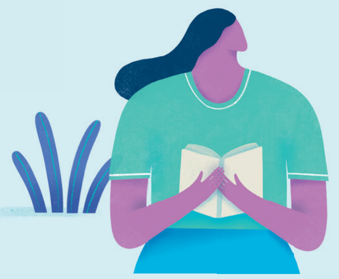

3 Toma tu muestra de sangre

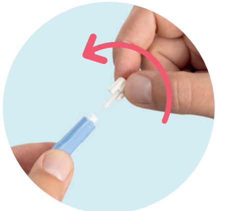

Lee los consejos para la toma de muestras de sangre. Gira la tapa blanca de la lanceta para retirarla. No la jales. Límpiate el dedo con el hisopo con alcohol y déjalo secar al aire.

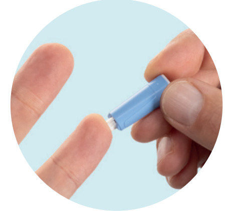

Cuando estés listo, coloca la lanceta en un lado de la yema del dedo. Presiona firmemente hacia abajo hasta que escuches un clic.

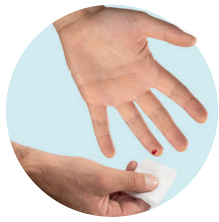

Usa la gasa para limpiar la primera pequeña gota de sangre.

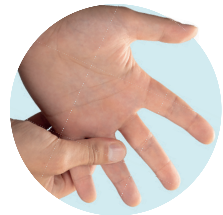

Masajea la mano y el dedo. No toques ni aprietes la yema del dedo; hacerlo obstruye el flujo sanguíneo.

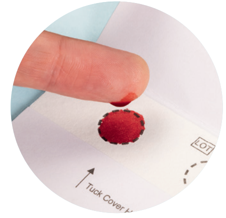

Vuelca la sangre en un círculo y llénalo completamente como se muestra. Tanto la parte frontal como la trasera del círculo se verán completamente rojas cuando esté completo. Repite para el segundo círculo. Cubre por completo al menos un círculo.

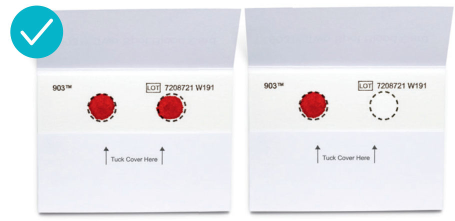

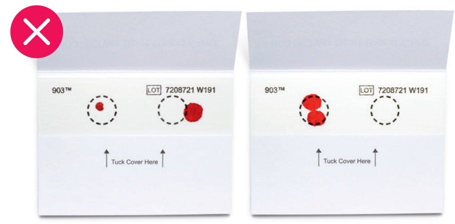

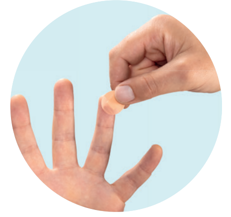

Colócate el apósito en el dedo.

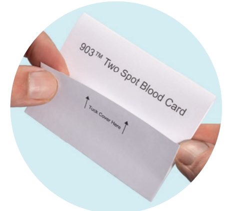

Dobla la cubierta de la tarjeta de toma muestra de sangre e insértala con cuidado debajo de la solapa (marcada con flechas). No aplanes ni apliques presión sobre la tarjeta.

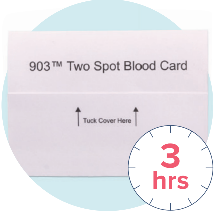

Deja secar la tarjeta de toma de muestras de sangre durante 3 horas. No apliques calor para ayudar a secar.

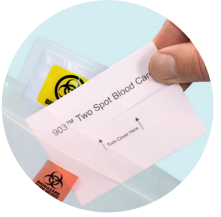

Coloca la bolsa de envío de la muestra de sangre y el tubo de transporte de la muestra de orina en la bolsa para residuos biológicos peligrosos y sella la bolsa.

4 Empaca las muestras y envíalas

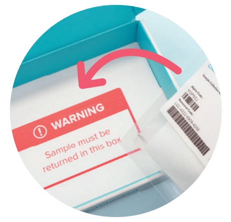

Coloca la bolsa para residuos biológicos peligrosos en la caja del kit. Verifica que hayas completado la etiqueta de la bolsa para residuos biológicos peligrosos.

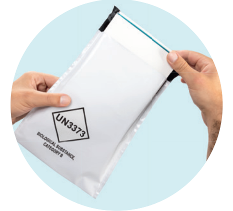

Coloca la caja del kit en el sobre de envío proporcionado.

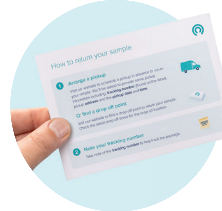

Envía el kit el mismo día. Revisa la tarjeta de entrega en la caja de tu kit para ver el método de envío.

Contenido del kit

Verifica tener todos estos artículos en tu kit:*

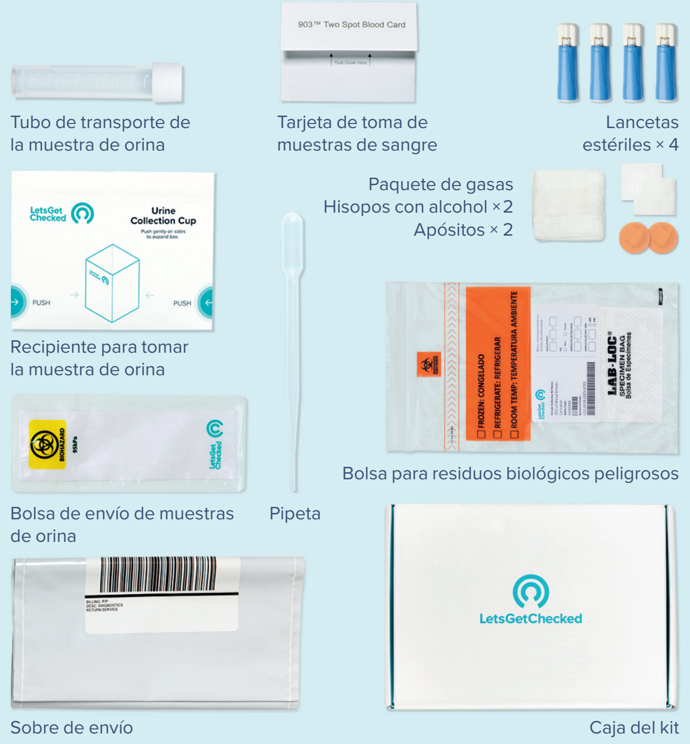

*El aspecto o el color de los artículos pueden ser diferentes de lo que se ve en estas imágenes.

LGC_ACC_BLOOD_DBS_AND_URINE_MID_QR_IFU_111_R0_PREACTIVATED_US_6_PG_WITH_SPANISH_V5.indd 13-14
07/09/2023 10:01
