# Supplementary material for: Development and validation of an LC-MSMS method to quantify creatinine from dried blood spots
Source: J Mass Spectrom Adv Clin Lab. 2024 Mar 6;32:50–9. doi: 10.1016/j.jmsacl.2024.03.001 (PMC10950697; doi:10.1016/j.jmsacl.2024.03.001)
Supplement: Supplementary data 2 [file mmc2.pdf]

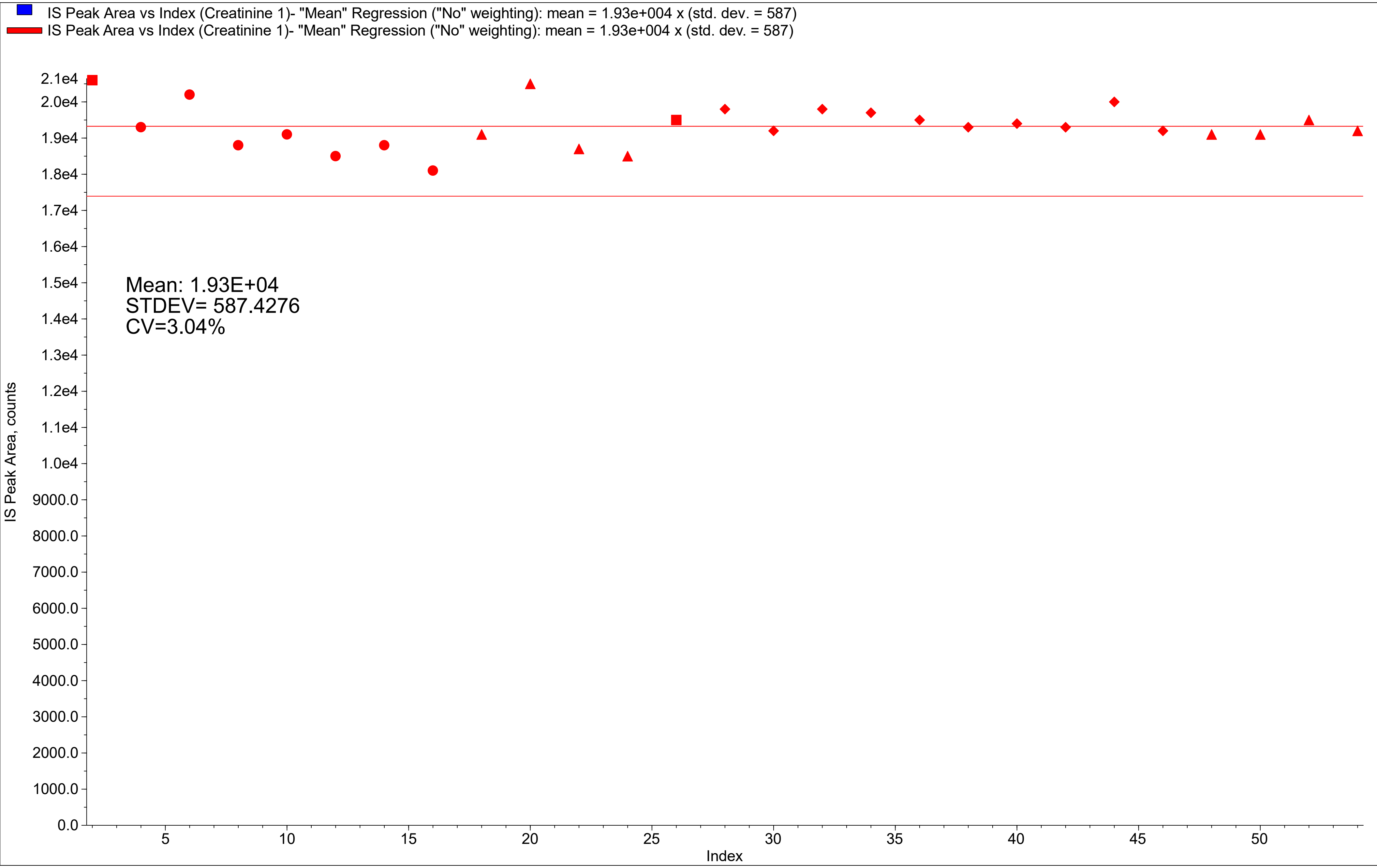

IS Peak Area vs Index (Creatinine 1)- "Mean" Regression ("No" weighting): mean = 1.93e+004 x (std. dev. = 587)
IS Peak Area vs Index (Creatinine 1)- "Mean" Regression ("No" weighting): mean = 1.93e+004 x (std. dev. = 587)
Mean: 1.93E+04
STDEV= 587.4276
CV=3.04%
IS Peak Area, counts
Index
